# Nivolumab-Induced Myasthenia Gravis Concomitant With Myocarditis, Myositis, and Hepatitis

**DOI:** 10.7759/cureus.18040

**Published:** 2021-09-17

**Authors:** Sawyer J Bawek, Ryan Ton, Margaret McGovern-Poore, Bilal Khoncarly, Ravish Narvel

**Affiliations:** 1 Internal Medicine, Lake Erie College of Osteopathic Medicine - Bradenton, Jacksonville, USA; 2 Internal Medicine, Lincoln Memorial University DeBusk College of Osteopathic Medicine, Jacksonville, USA; 3 Internal Medicine, Ascension St. Vincent's Riverside, Jacksonville, USA

**Keywords:** pleural effusion, melanoma, hepatitis, myositis, myocarditis, myasthenia gravis, nivolumab

## Abstract

We report a case of myasthenia gravis, myocarditis, and myositis following the treatment of melanoma with nivolumab. The patient was a 68-year-old Caucasian male with stage 3 melanoma status after two doses of nivolumab with shortness of breath, intermittent palpitations, dizziness, and nausea. During his initial evaluation, he was found to have atrial fibrillation with rapid ventricular response along with new-onset proximal muscle weakness, double vision, dysphagia, and ptosis of the right eye. Further diagnostic workup of the pleural effusion with CT of the chest showed large right pleural effusion with adjacent atelectasis. Thoracentesis was completed without complications and resulted in an exudative effusion with negative cytology and cultures. Serologic studies showed elevated troponin and serum creatine kinase, negative acetylcholine receptor antibody, and negative modulating antibody. Despite negative antibody tests, the patient's symptoms suggested a clinical diagnosis of myasthenia gravis. The ice pack test was performed, which showed temporary improvement of the patient's ptosis. Given the suspicion for myasthenia gravis and positive ice pack test, he was treated with corticosteroids, intravenous immunoglobulin (IVIG), and pyridostigmine. He completed a total of three doses of IVIG with improvement in diplopia. Despite steroids and respiratory support with BiPAP (bilevel positive airway pressure), on the 14th day of hospitalization, the patient had multiple organ failure along with worsening respiratory failure. The patient discussed the situation with his family, and they decided on hospice care. The patient was discharged to hospice on admission day 14.

## Introduction

Nivolumab is a human IgG4 monoclonal antibody that binds to programmed cell death-1 (PD-1) receptors and blocks the interaction with programmed death-ligand 1 (PD-L1) and programmed death-ligand 2 (PD-L2). This serves as a checkpoint that blocks PD-L1 and PD-L2 from binding to T-cells, which block inhibitory ligand-receptor interactions, thus facilitating T-cell activation and killer T-cell activity. Nivolumab and other medications in its class, such as pembrolizumab and ipilimumab, are used in several types of cancer, including melanoma and non-small cell lung cancer [1-3]. Known side effects of nivolumab include hepatitis, pneumonitis, acute renal failure, endocrine disorders, and other immune-related adverse events (irAEs) [1-4]. Despite the clinical benefits of nivolumab, immune-related adverse effects are related and can be fatal. Multiple cases of myasthenia gravis, myocarditis, and myositis were reported following the start of nivolumab [5-7]. We present the case of a 68-year-old male with metastatic neuroendocrine carcinoma and stage 3 melanoma who developed myasthenia gravis, myositis, and myocarditis three weeks following the second dose of nivolumab treatment.

## Case presentation

A 68-year-old Caucasian male with a history of metastatic neuroendocrine carcinoma and stage 3 melanoma presented to the ED with three weeks of shortness of breath. Three weeks prior to his hospitalization, nivolumab was initiated for the treatment of melanoma. The patient received a total of two doses of nivolumab prior to admission. He was found to have a left bundle branch block (LBBB) on electrocardiogram (ECG), a normal brain natriuretic peptide level, and an elevated troponin level of 2.16 ng/mL (normal: <0.04 ng/mL), which was felt to be a type 2 NSTEMI (non-ST segment elevation myocardial infarction). An echocardiogram revealed an ejection fraction of 55-60% with wall motion abnormality consistent with his underlying bundle branch block. A left heart catheterization was performed, which showed normal coronaries and valvular function. A CT of the chest demonstrated a large right pleural effusion with adjacent passive atelectasis. He underwent a thoracentesis with the removal of 1,375 mL of fluid. Analysis of the pleural fluid resulted in an exudative effusion with negative cytology and cultures. At this point, the patient was deemed stable enough to be discharged home with home healthcare.

**Figure 1:**
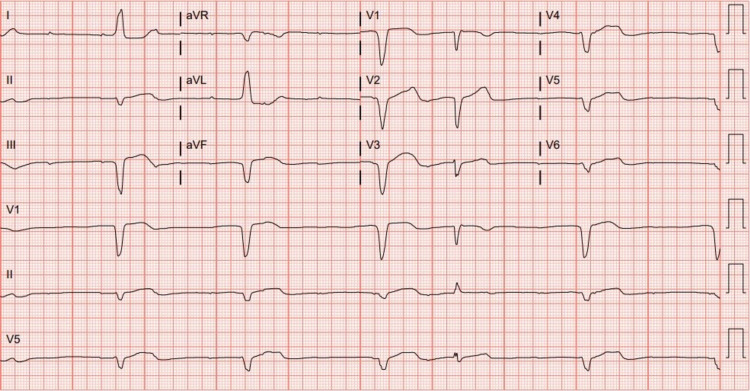
Electrocardiogram showing a heart rate in the 30s with third-degree atrioventricular block and junctional escape with a left bundle branch block

The patient was readmitted four days later after he developed worsening shortness of breath and near syncope. An ECG showed a heart rate in the 30s with third-degree atrioventricular block and junctional escape with an LBBB (Figure 1). CT of the brain was negative for acute infarct or hemorrhage. Repeat chest X-ray demonstrated persistent small bilateral pleural effusions with stable atelectasis. Labs on admission showed a troponin that was again elevated but was now more elevated at 11.09 ng/mL. His liver function enzymes were also found to be significantly more elevated on this admission, with aspartate transaminase (AST) of 537 U/L (normal: 5-34 U/L), alanine transaminase (ALT) of 315 U/L (normal: 0-55 U/L), and alkaline phosphatase of 167 U/L (normal: 44 to 147 U/L). The patient had a gamma-glutamyl transferase (GGT) level that was within normal limits, prompting further investigation into causes of elevated liver enzymes including ischemia and medication-induced hepatitis. Repeat thoracentesis again showed an exudative effusion with negative cytology and cultures. An emergent Medtronic dual-chamber pacemaker was placed secondary to his complete heart block.

On admission day 2, the patient was found to have new-onset diplopia, dysphagia, and ptosis of the right eye. Neurological examination demonstrated horizontal and binocular diplopia occurring in all directions and generalized weakness. Treatment was initiated with pyridostigmine 60 mg three times a day and intravenous immunoglobulin (IVIG) for an acute exacerbation of presumed myasthenia gravis, and serological studies were sent including acetylcholine binding, blocking, and modulating antibodies, as well as a striated muscle IgG antibody, which all returned negative. It was also apparent that his elevated creatine kinase and peaking troponin levels were also diagnostic for myositis given his proximal muscle weakness and myocarditis given his poor cardiac function, heart block, and elevated troponin levels. He developed intractable diarrhea presumed to be secondary to the IVIG, which was discontinued after three doses. On admission day 6, the patient experienced episodes of paroxysmal atrial fibrillation and an episode of supraventricular tachycardia with heart rate in the 200s. The next day, the patient’s respiratory function declined to acute hypercapnic respiratory failure requiring bilevel positive airway pressure (BiPAP). Due to his severely decompensated state, 1,000 mg of methylprednisolone (mPSL) IV infusion was initiated on admission day 8, which was to be repeated daily for a duration of three days. The course of mPSL was followed by 100 mg prednisone taper. The patient’s symptoms initially improved following the mPSL pulse therapy; however, his oxygen requirement later increased to BiPAP 100% FiO_2_. He eventually progressed to multiple organ failure and possible heparin-induced thrombocytopenia as his platelets dropped significantly since admission. Ultimately, the patient and family decided to transfer to hospice care.

## Discussion

Improved survival rates in patients with melanoma have been shown in immune checkpoint inhibitors that target programmed cell death protein-1 and cytotoxic T-lymph associated protein-4; however, potentially life-threatening adverse effects have also been identified [8]. These include hepatotoxicity, myositis, myasthenia gravis, and myocarditis. Given the patient’s normal liver enzymes prior to initiation of immunotherapy, normal GGT, and negative hepatitis panel, he was presumed to have medication-induced hepatitis. The combination of myasthenia gravis with myocarditis is known as Herzmyasthenie. This association between myasthenia gravis and myocarditis was first described in 1901 by a German Physician named Leopold Laquer where he described the case of a 30-year-old man with myasthenia gravis symptoms (unilateral ptosis, difficulties in deglutition, and weakness of the arms) and symptoms of myocarditis. Laquer thought that the muscular disorder had spread to the heart muscle causing a "myasthenia of the heart." It has been reported that myasthenia gravis occurs in 0.12% of patients being treated with nivolumab. Patients who develop myasthenia gravis have been reported to have a 16-37% chance of also having myocardial changes [9]. The diagnosis of myocarditis is thought to be underdiagnosed in patients with myasthenia gravis. The most common type of cancer for irAEs is melanoma (48%) [10]. It has been reported that the mean duration of treatment for patients is 54 days [8]. Our 68-year-old Caucasian male’s bulbar, diplopia, fatigue, and respiratory failure symptoms failed on initial IV Decadron® and three doses of IVIG. Previous case reports have shown improvement of myocarditis when treated with 1,000 mg of IV mPSL for three days followed by 1 mg/kg of mPSL taper. The patient’s respiratory failure was rapidly progressing. Stress dose steroids of 1,000 mg of mPSL were given on day 8 of admission. To the best of our knowledge, this is the latest stress dose steroid that has been started in patients with concurrent myasthenia gravis, myocarditis, and myositis. The delayed initiation of stress dose steroids was due to the inability to diagnose myocarditis definitively. The patient’s recent emergent placement of the pacemaker was a contraindication for MRI, and the patient’s unstable condition prevented confirmatory biopsy. This patient was eventually treated for presumed myocarditis, which was diagnosed clinically. Following completion of mPSL for three days, the patient had improvement of his respiratory status. Prednisone 100 mg taper was initiated as indicated. He continued treatment with pyridostigmine 60 mg three times a day. The patient was able to carry out a conversation and reported improvement. He remained on BiPAP with FiO_2_ 60% with multiple attempts to wean off to Oxymizer® 8 L. After three days, his clinical status declined, requiring BiPAP with FiO_2_ 100% and having concurrent multiple organ failure. The patient and his family ultimately decided to proceed with hospice care on hospital day 14. He was discharged to inpatient hospice at that time.

## Conclusions

We reported a case of concurrent myasthenia gravis, myocarditis, myositis, and hepatitis following treatment for stage 3 melanoma with nivolumab. Studies have shown that steroid dosage of less than 1,000 mg of mPSL is insufficient to treat medication-induced myocarditis in patients with toxicity to nivolumab. Although mPSL pulse therapy can exacerbate the myasthenia gravis, priority should be placed on treating the myocarditis due to the higher mortality rate. Further studies are needed to establish the optimal timing and approach to diagnosing and managing these potential life-threatening complications of nivolumab toxicity.
